# Supplementary material for: Knowledge management process, knowledge based innovation: Does academic researcher’s productivity mediate during the pandemic of covid-19?
Source: PLoS One. 2021 Dec 22;16(12):e0261573. doi: 10.1371/journal.pone.0261573 (PMC8694422; doi:10.1371/journal.pone.0261573)
Supplement: S1 Appendix — (DOCX) [file pone.0261573.s001.docx]

**Appendix**

**Knowledge creation:**

**KC 1:** We have an explicit strategy for knowledge development during the pandemic of COVID-19.

**KC 2:** In our university, we agree on how we should get new knowledge during the pandemic of COVID-19.

**KC 3:** We are stimulated to acquire and/or develop new knowledge during the pandemic of COVID-19.

**KC 4:** We have developed ways to support the creation of new knowledge (e.g. traineeships and job rotation) during the pandemic of COVID-19.

**KC 5:** We know how to innovate during the pandemic of COVID-19.

**KC 6:** We focus on learning and exploring new ways of working during the pandemic of COVID-19.

**KC 7:** We have the right systems to capture and share new ideas and experiences during the pandemic of COVID-19.

**KC 8:** I like to learn during the pandemic of COVID-19.

**KC 9:** I always effectively develop new knowledge when I need it during the pandemic of COVID-19.

**Knowledge Acquisition**

**KA 1:** Knowledge is obtained from researchers in the university during the pandemic of COVID-19.

**KA 2:** Knowledge is obtained from partners during the pandemic of COVID-19.

**KA 3:** Knowledge is obtained from the employees of concerned department during the pandemic of COVID-19.

**KA 4:** Our University uses feedback from projects to improve subsequent projects during the pandemic of COVID-19.

**KA 5:** Our University has processes for acquiring knowledge about inventions during the pandemic of COVID-19.

**KA 6:** Our University has processes for acquiring knowledge about new products/services within industry during the pandemic of COVID-19.

**KA 7:** Our University has processes for acquiring knowledge about competitors within industry during the pandemic of COVID-19.

**Knowledge sharing:**

**KS 1:** The strategy of our university can only be realised if knowledge is shared during the pandemic of COVID-19.

**KS 2:** In our university “knowledge sharing is power” applies more than “possessing knowledge is power” during the pandemic of COVID-19.

**KS 3:** We spend time to share our ideas and experiences with others, even if this is not directly relevant for our job during the pandemic of COVID-19.

**KS 4:** We know how we can optimally share our knowledge with each other during the pandemic of COVID-19.

**KS 5:** The management motivates staff to share knowledge by building trust, giving incentives, making available time and resources during the pandemic of COVID-19.

**KS 6:** We have the right systems, like databases, intranets, team-rooms and e-mail to support knowledge sharing during the pandemic of COVID-19.

**KS 7:** The way we are organized (departments, meetings) doesn’t introduce any barriers for knowledge sharing during the pandemic of COVID-19.

**KS 8:** I like to share my ideas and experiences with others during the pandemic of COVID-19.

**KS 9:** By sharing my knowledge I have made a significant contribution to the university during the pandemic of COVID-19.

**Knowledge utilization:**

**KU 1:** We have a systematic approach to make optimal use of knowledge in our business processes during the pandemic of COVID-19.

**KU 2:** We know how we can use the available knowledge in our work during the pandemic of COVID-19.

**KU 3:** We apply knowledge to improve and to innovate in our job during the pandemic of COVID-19.

**KU 4:** We are encouraged to make use of the knowledge that is available during the pandemic of COVID-19.

**KU 5:** We agree on how we can make optimal use of our knowledge during the pandemic of COVID-19.

**KU 6:** We know how to link knowledge to the organization processes and activities during the pandemic of COVID-19.

**KU 7:** We have systems that make it easier to make use of available knowledge during the pandemic of COVID-19.

**KU 8:** I am flexible in applying other people’s knowledge, in order to become more efficient, effective etc, during the pandemic of COVID-19.

**KU 9:** I prefer to use other people’s ideas and suggestions, instead of figuring it out for myself during the pandemic of COVID-19.

**Knowledge-worker productivity**

**Research autonomy at work**

**RAW 1:** The working environment allows me to make decisions about what methods I use to complete my work during the pandemic of COVID-19.

**RAW 2:** The working environment gives me considerable opportunity for independence and freedom in how I do the work during the pandemic of COVID-19.

**RAW 3:** The working environment allows me to decide on my own how to go about doing my work during the pandemic of COVID-19.

**Meeting time demands**

**MTD 1:** I work in required hours during the pandemic of COVID-19.

**MTD 2:** I work without breaks or rests during the pandemic of COVID-19.

**MTD 3:** I put in extra hours to keep up my work during the pandemic of COVID-19.

**Work efficiency**

**WE 1:** I wisely utilize the resources to produce the required output during the pandemic of COVID-19.

**WE 2:** I use minimum resources to achieve desired satisfaction during the pandemic of COVID-19.

**WE 3:** I complete my tasks before the required time frame during the pandemic of COVID-19.

**Innovation**

**Product Innovation**

**PI 1:** Our university is often first to introduce new research idea during the pandemic of COVID-19.

**PI 2:** Our new research articles often perceived very novel by readers during the pandemic of COVID-19.

**PI 3:** Our university has published more innovative research articles during the last few years as compared to others during the pandemic of COVID-19.

**PI 4:** The technological equipments in our labs are very up to date during the pandemic of COVID-19.

**PI 5:** Our university launches new research projects as per the community requirements during the pandemic of COVID-19.

**Process Innovation**

**PRI 1:** Our university imports new process technology during the pandemic of COVID-19.

**PRI 2:** Our university obtains process technology patents during the pandemic of COVID-19.

**PRI 3:** Our university adopts advanced real-time process control technology during the pandemic of COVID-19.

**PRI 4:** Our university imports advanced automatic quality restriction equipment/software during the pandemic of COVID-19.

**Problems solving Capabilities**

**PSC 1:** We determine how to solve a task before begin during the pandemic of COVID-19.

**PSC 2:** We check how well we are doing when we solve a task during the pandemic of COVID-19.

**PSC 3:** We carefully plan our course of actions during the pandemic of COVID-19.

**PSC 4:** We judge the correctness of our work during the pandemic of COVID-19.

**PSC 5:** I am confident I can understand the most complex material presented by a group member during the pandemic of COVID-19.

**Services Innovation**

**SI 1:** Our university has developed service that is new to the researchers during the pandemic of COVID-19.

**SI 2:** Our university has developed service that is new to the departments during the pandemic of COVID-19.

**SI 3:** Our university has engaged to improve existing service during the pandemic of COVID-19.

**SI 4:** Our university has introduced processes to enhance researchers access to our service during the pandemic of COVID-19.

**Radical Innovation**

**RI 1:** Compared with competitors, our university introduces new products that are more radically new to the researchers (e.g., adopting new technology) during the pandemic of COVID-19.

**RI 2:** Compared with competitors, our university introduces new products that offer more radical features (e.g., adopting new design or new elements) during the pandemic of COVID-19.

**RI 3:** Compared with competitors, our university introduces new products that require radically changes in the researcher’s way of using them during the pandemic of COVID-19.
