# Supplementary material for: Knowledge management process, knowledge based innovation: Does academic researcher’s productivity mediate during the pandemic of covid-19?
Source: PLoS One. 2021 Dec 22;16(12):e0261573. doi: 10.1371/journal.pone.0261573 (PMC8694422; doi:10.1371/journal.pone.0261573)
Supplement: Attachment — Submitted filename: Response to Reviewers.docx [file pone.0261573.s002.docx]

Response to Reviewers

Dear Reviewers and Editors,

Thank you very much for the review of our manuscript, your time, and providing interesting guidelines, and comments on our paper…..we tried our best to address all the issues of our manuscript as per your respected suggestions.

Please the responses to your respected comments are as follows:

1. The manuscript ensured the PLOS ONE’s style requirements and formatted accordingly.
2. This study has adopted and adapted the questionnaire and all the relevant references are included in Table 1
3. The authors received no specific funding for this work.
4. References of Figures are highlighted for the said purpose
5. There are only two figures in the manuscript
6. All the tables are referred in the text and highlighted
7. All the references are according to the journal format and ensured corrections

Reviewers Comments

1. The design, population, and sample size of the study are cleared
2. The study was conducted in the setting of COVID-19 pandemic and the illustration, justification is present in the second paragraph of introduction.
3. Please the manuscript was edited by an expert editor
4. The methodology portion is now more clear and concise
5. Please the results are analyzed and explained properly
6. Please, in the conclusion, the aim of the study was to evaluate the effects of the knowledge management process on knowledge-based innovation along with mediating role of academic researchers’ productivity during the Pandemic of COVID-19 at Malaysia
7. Please the policy makers, practitioners, and researchers outside Malaysia can get advantage of the study results in term of knowledge management process to enhance the knowledge based innovation.
